# A Pilot Case-Control Study of Muscle and Bone Health Status in Patients Who Have Recovered From COVID-19 Infection

**DOI:** 10.7759/cureus.76283

**Published:** 2024-12-23

**Authors:** Shital Bhor, Dipali Ladkat, Nikhil Shah, Ketan Gondhalekar, Piyush Chaudhari, Anuradha Khadilkar

**Affiliations:** 1 Department of Growth and Pediatric Endocrinology, Hirabai Cowasji Jehangir Medical Research Institute, Pune, IND; 2 Department of Pediatric Endocrinology, MRR Children’s Hospital, Mumbai, IND; 3 Department of Infectious Disease, Jehangir Hospital, Pune, IND; 4 Department of Health Sciences, Savitribai Phule Pune University, Pune, IND

**Keywords:** ace-2 receptors, covid-19, handgrip strength, indian, lean mass

## Abstract

Background: Coronavirus disease 2019 (COVID-19), resulting from the novel severe acute respiratory syndrome coronavirus 2 (SARS-CoV-2), affects various bodily systems, including the heart, central nervous system, muscles, and bones, all of which harbor angiotensin-converting enzyme 2 (ACE-2) receptors similar to those in the respiratory system. However, research on the inflammatory response and its impact on systems such as the musculoskeletal one is relatively scarce. Our study aimed to investigate bone and muscle metrics as well as handgrip strength in individuals who recuperated from COVID-19 infection.

Methods: The pilot case-control study was conducted from June 2021 to September 2022, involving 25 adult patients aged 18-60 years who had recovered from COVID-19 infection, alongside 25 age- and gender-matched controls. Participants were recruited from a tertiary care hospital in Pune, and data on demographics, anthropometry, medical history, diet, and physical activity were recorded using standardized questionnaires. Dual-energy X-ray absorptiometry (DXA) and peripheral quantitative computed tomography (pQCT) were used to assess bone and muscle parameters, whereas a handgrip dynamometer was used to measure muscle strength. Statistical analysis was performed using SPSS version 26.0 (IBM Corp., Armonk, NY).

Results: Post-COVID-19 recovered female patients exhibited lower lean mass, muscle density, and handgrip strength, alongside higher body fat levels.

Conclusion: Lean mass, muscle density, and handgrip strength were lower and body fat was higher in post-COVID-19 recovered females, thus indicating the need to focus on improving the musculoskeletal health of females post-hospitalization due to a serious illness such as COVID-19 infection.

## Introduction

Coronavirus disease 2019 (COVID-19) is caused by the novel severe acute respiratory syndrome coronavirus 2 (SARS-CoV-2), and it has affected and has been changing people's lives around the world since 2020. As previously demonstrated in the case of other coronaviruses, SARS‐CoV‐2 can infect different systems that share the same angiotensin-converting enzyme 2 (ACE‐2) receptors in the respiratory system. Therefore, most of the extrapulmonary manifestations due to COVID-19 infection occur in the organs or systems with cells that express ACE‐2 receptors (i.e., the heart, central nervous system, and muscle, among others) [1].

COVID-19 affects multiple organs, including the musculoskeletal system, and can cause symptoms such as fatigue, joint pain, muscle pain, and muscle weakness. These symptoms may persist for weeks or even months after the infection has resolved [2]. The virus is also believed to enter host cells via ACE-2 receptors that affect bone remodeling, leading to osteopenia or osteoporosis, which is characterized by low bone mineral density (BMD) [3].

COVID-19 infection has also been linked to high levels of inflammatory cytokines, hospitalization, and prolonged immobilization. Further, corticosteroid treatment is often administered for serious comorbidities, which increase the risk of bone loss and resorption [4].

A previous study reports that during the COVID-19 infection outbreak, patients who were treated with corticosteroids showed lower bone mineral density (BMD) in comparison to healthy controls, in the long-term [5].

The inflammatory response to SARS-CoV-2 and its effect on the respiratory system has been extensively studied. However, there is a lack of sufficient literature addressing the inflammatory response and its effects on other organ systems, especially the musculoskeletal system. Thus, our pilot study aimed to assess bone and muscle parameters and handgrip strength among patients who had recovered from COVID-19 infection. We specifically studied individuals who had undergone hospitalization and corticosteroid therapy for the treatment of COVID-19 infection. We hypothesized that there would be no difference in the bone and muscle health status of patients who had recovered from COVID-19 infection in comparison with healthy age- and gender-matched controls. Thus, our primary objective was to compare bone and muscle health status (as measured by dual-energy X-ray absorptiometry (DXA) and peripheral quantitative computed tomography (pQCT)) in patients who have recovered from COVID-19 infection in comparison with healthy age- and gender-matched controls. Our secondary objective was to compare muscle strength in patients who had recovered from COVID-19 infection with healthy age- and gender-matched controls using the JAMAR PLUS hand dynamometer (Patterson Medical, Sammons Preston, Jackson, MI).

## Materials and methods

The study design was a pilot case-control study performed between June 2021 and September 2022. A pilot case-control study is cost-effective, time-efficient, feasible, and well-suited for generating a hypothesis. We assessed 25 adult patients aged 18-60 years who had recovered from COVID-19 infection and 25 age- and gender-matched healthy controls. Controls were screened for COVID-19 symptoms as well as other muscle and bone disorders. Study participants (cases and controls) were enrolled from a tertiary-level care hospital in Pune, Western India after they gave written informed consent and satisfied inclusion and exclusion criteria. An ethics committee approval was obtained from the institutional ethics committee (approval date: 11-07-2022) before any study procedures were performed, and all participants gave written informed consent.

Inclusion criteria

Patients who were diagnosed with COVID-19 infection confirmed by a positive throat swab test by real-time reverse transcription-polymerase chain reaction (RT-PCR) along with the history of steroid treatment prescribed during hospitalization or post-discharge from hospitalization and had undergone hospitalization 2-14 months before enrolment were included in the study. Healthy participants were recruited from subjects coming to the center for routine health checks. Participants willing to provide a signed informed consent for the study were enrolled.

Exclusion criteria

Patients with rheumatoid arthritis, systemic lupus erythematosus, or any other bone, joint, or muscle disease and with known osteoporosis were excluded from the study. Patients with any other chronic disorder likely to affect muscle or bone such as uncontrolled diabetes were also excluded. Finally, participants who did not give informed consent were also excluded from the study.

Data on demography and medical details including duration of admission, past medical history, treatment given during admission, history of steroids during and after discharge from the hospital, current symptoms, six-minute walk test, and use of oxygen and ventilator were collected using a standardized questionnaire. The medical history provided by patients was verified from hospital medical records. Two-day diet recall and physical activity details were recorded by trained nutritionists. The protein estimated average requirement (EAR) was calculated using the Indian Council of Medical Research-National Institute of Nutrition (ICMR-NIN) guidelines [6].

Anthropometry

The standing height of patients and healthy participants was measured using a portable stadiometer (Leicester Height Meter, Child Growth Foundation, UK) to the nearest millimeter, and weight was measured using an electronic scale to the nearest 100 g. Body mass index (BMI) was computed by dividing weight in kilograms by height in meters square.

DXA measurements

DXA scan measures bone mineral density, lean mass, and fat mass percentage. Areal BMD (aBMD) was measured using a DXA scan using the Lunar iDXA (GE Healthcare, WI) fan beam scanner (encore software version 16). The machine underwent daily calibration, with service engineers routinely reviewing the calibration data. The same operator conducted all scans and analyses. The coefficient of variation (CV) for L1-L4 areal BMD and L1-L4 bone mineral content (BMC) was 1% and 2.8%, respectively. The sites measured were the lumbar spine, femur, and total body bone mineral content (bone mineral content (BMC) (g), areal bone mineral density (aBMD) (g/cm^3^), and bone area (BA) (cm^2^)). As per the World Health Organization classification for osteoporosis, a participant with a negative T-score (T-score is a comparison of the patient's bone density with healthy, young individuals of the same sex) of -2.5 or less was classified as having osteoporosis [7]. Muscle mass was assessed using appendicular skeletal muscle mass index (ASMI), which was calculated as the sum of lean mass at arms and legs in kilograms divided by the square of height in meters [8].

Peripheral quantitative computed tomography (pQCT)

Peripheral quantitative computed tomography (pQCT) measurements of the radius were conducted using the Stratec XCT 2000 device (Stratec Inc., Pforzheim, Germany) and analyzed with the integrated Stratec 2000 software version 6.2. pQCT measures trabecular density, cortical density, muscle density, and cortical, periosteal, and endosteal thickness. For radius measurements, the distance between the ulnar styloid process and the olecranon was determined on the subject's non-dominant hand using a non-stretchable measuring tape for the measurement of the ulna. In the scout view, a reference line was positioned through the center of the ulnar border of the articular cartilage. Scans were acquired at 4% and 66% of the radial length, as defined by this measurement [9,10]. At the 4% site, images were obtained using a voxel size of 0.59 mm and a slice thickness of 2.5 mm, contour mode 2, peel mode 2, and a threshold of 180 mg/cm³ for trabecular volumetric bone mineral density (vBMD). Cortical vBMD and Stress-Strain Index (SSI) were measured at 66% of the radius using a threshold of 711 mg/cm^3^, contour mode 3. The coefficient of variation (CV) for trabecular and cortical density was 3.0% and 0.8%, respectively.

Scans of the non-dominant tibia were performed with a voxel size of 0.5 mm and a slice thickness of 2 mm, targeting sites located at 4% and 66% of the tibial length proximal to the distal endplate. The CT scanning speed was 30 mm/s, with a scout view speed of 40 mm/s. Data were analyzed using Stratec XCT software version 6.2. For the 4% site, the calculated bone density (CALCBD) analysis applied contour mode 1 and peel mode 1, with a threshold of 180 mg/cm³. Trabecular bone was defined as the inner 45% of the total cross-sectional area (Tot.A). The measured parameters included total volumetric bone mineral density (Tot.vBMD) and trabecular volumetric bone mineral density (Tb.vBMD). Stress-Strain Index (SSI), an estimate of bone strength, was obtained at a threshold of 280 mg/cm^3^ using cortmode [11].

Handgrip strength

To assess muscle function, handgrip strength was measured using a digital JAMAR PLUS hand dynamometer (Patterson Medical, Sammons Preston, Jackson, MI). The maximal isometric grip strength of the non-dominant hand was measured as follows. Participants held the dynamometer with a flexed elbow, and care was taken to ascertain that it did not touch the trunk. Participants were then asked to exert maximal force on the dynamometer, and the maximum value of three trials (with a 30-second rest between the trials) was noted. To avoid the influence of the circadian cycle, all measurements were performed in the morning.

Statistical analysis

All statistical analyses were carried out using the SPSS for Windows software program version 26 (IBM Corp., Armonk, NY). All outcome variables were tested for normality before performing statistical analyses. Non-parametric tests were used as data was not normally distributed. Differences in means were tested using the Mann-Whitney U test for non-parametric data. P<0.05 was considered statistically significant. Data are represented in terms of median and interquartile range (IQR).

## Results

A total of 25 patients who had recovered from COVID-19 infection and 25 healthy age- and gender-matched controls were included in this study. The average duration of hospitalization of COVID-19-infected patients was 10 days. The average total steroid dose taken by patients was 3.7 mg/kg body weight. Most patients were on injection methylprednisolone (steroid) 40 mg twice a day for five days. Of the patients, 40% had required oxygen therapy during hospitalization. The six-minute walk test that was performed in patients showed the same oxygen saturation in pre- and post-test, i.e., 98%. The pre-walk test pulse was 84 among post-COVID-19 recovered patients, and the post-walk test pulse was 86. Only 8% of the patients were on ventilators during hospital admission. Post-COVID-19 patients reported symptoms even after recovery from COVID-19 infection, namely, generalized weakness, myalgia, arthralgia, breathlessness, loss of appetite, interrupted sleep, emotional distress, and hyperacidity. At the time the data were collected, there were no significant differences in physical activity and estimated average intake of protein among post-COVID-19 recovered patients and controls (p>0.05).

Anthropometric, DXA, pQCT at radius and tibia, and handgrip strength parameters of the study subjects are illustrated in Table 1. There were no statistically significant differences between male patients among any of the parameters; in female patients, there were no differences between the two groups with respect to age, anthropometric parameters, diet, and physical activity (p>0.05 for all). Further, aBMD at the lumbar spine, femur T-scores, and appendicular skeletal muscle index were also not significantly different between female patients of the two groups. However, lean mass percentage was significantly lower in post-COVID-19 recovered female patients than in their healthy age- and gender-matched counterparts (p<0.05). Further, fat percentage was significantly higher among post-COVID-19 recovered female patients than in healthy female controls. Muscle density at the tibia in the post-COVID-19 recovered female patients was significantly lower than in the controls (p<0.05). Handgrip strength in female patients who had recovered from COVID-19 infection was also significantly lower than in healthy female patients (p<0.05). Further, female patients were analyzed as per the history of menopause. Post-COVID-19 recovered female patients who had not yet reached menopause had lower muscle density compared to healthy women who also had not reached menopause (p<0.05).

**Table 1:** Demographic, clinical, bone, and muscle parameters of the study population (N=50) Values are expressed as median (interquartile range). *Statistically significant COVID-19: coronavirus disease 2019, pQCT: peripheral quantitative computed tomography, DXA: dual-energy X-ray absorptiometry, EAR: estimated average requirement, L1-L4 BMD: lumber 1 to lumber 4 vertebrae bone mineral density, BMI: body mass index

Serial number	Parameters	Post-COVID-19 recovered male patients (n=15)	Healthy male patients (n=14)	p-value	Post-COVID-19 recovered female patients (n=10)	Healthy female patients (n=11)	p-value
1	Age (years)	44.0 (12.7)	46.4 (15.2)	0.88	44.4 (22.5)	41.7 (20.3)	0.91
2	Height (cm)	169.6 (7.3)	171.8 (5.2)	0.29	156.3 (7.1)	151.5 (8.4)	0.60
3	Weight (kg)	74.2 (9.2)	66.9 (15.2)	0.35	67.3 (12.0)	61.9 (8.2)	0.03*
4	BMI (kg/m^2^)	25.4 (1.8)	28.1 (5.5)	0.17	23.6 (5.4)	25.2 (3.5)	0.06
5	Protein EAR (g)	130.2 (118.6)	100 (45.3)	0.27	75 (37.4)	94.4 (72.2)	0.46
6	Physical activity (weekly/minute)	840 (735)	2902 (3517)	0.20	1190 (420)	585 (2425)	0.60
	DXA scan parameters						
7	L1-L4_BMD (g/cm^3^)	1.1 (0.2)	1.2 (0.2)	0.42	1.1 (0.2)	1.1 (0.2)	0.75
8	L1-L4 T-score	-0.5 (1.4)	-0.3 (1.5)	0.47	-0.6 (1.8)	-0.4 (1.2)	0.75
9	Appendicular skeletal muscle index	8 (1)	7.6 (1.2)	1.00	6.7 (1.8)	7.1 (1.4)	0.70
10	Total T-score (femur)	-0.4 (1.2)	-0.5 (1.1)	0.42	-0.6 (1.6)	-0.3 (1.2)	0.75
11	Lean mass percentage	62.7 (3.6)	65.2 (17.6)	0.29	52 (48.3)	64.6 (19.8)	0.03*
12	Fat percentage	33.7 (2)	29.2 (7.8)	0.12	44.8 (1.9)	41 (6.5)	0.01*
	Radius pQCT parameters						
13	Trabecular density 4% (mg/cm^3^)	187.3 (62.5)	157.6 (49.2)	0.13	170.2 (52.5)	126.7 (67.6)	0.37
14	Cortical density 66% ( mg/cm^3^)	1144.6 (76.3)	1144.7 (30.3)	0.33	1169.6 (56.1)	1187.9 (96.3)	0.93
15	Stress-Strain Index 66% (mm^3^)	292.7 (92.1)	314.9 (87.9)	0.32	174.8 (96.1)	191.9 (36.2)	0.32
16	Muscle density 66% (mg/cm^3^)	74.3 (4.5)	74.6 (2.6)	0.51	73.2 (1.8)	74.2 (2.3)	0.04
17	Muscle area 66% (mm^2^)	6322 (1759.75)	6147 (2033.25)	0.07	4890 (1436.5)	5618 (1285.0)	0.59
18	Fat-to-muscle area ratio at 66%	40.3 (8.2)	27 (11.8)	0.07	77.7 (42.5)	61.6 (21.3)	0.06
19	Cortical thickness at 66% (mm)	2.3 (0.6)	2.2 (0.5)	0.91	1.9 (0.4)	2.2 (0.7)	0.18
20	Periosteal thickness at 66% (mm)	42.5 (4.4)	42.8 (4.5)	0.70	37.1 (3.9)	36.0 (3.9)	0.69
21	Endosteal thickness at 66% (mm)	26.0 (7.4)	27.9 (5.7)	0.80	24.7 (7.2)	23.5 (7.0)	0.69
	Tibia pQCT parameters						
22	Trabecular density 4% (mg/cm^3^)	214.0 (40.2)	197.6 (46.0)	0.13	196.5 (25.6)	171.2 (82.1)	0.59
23	Cortical density 66% (mg/cm^3^)	1141.9 (46.0)	1113.4 (49.9)	0.33	1153.0 (35.5)	1150.3 (62.8)	1.00
24	Stress-Strain Index 4% (mm^3^)	2655.3 (658.2)	2474.01 (827.8)	1.00	1894.5 (272.5)	1874.1 (346)	0.86
25	Muscle density 66% (mg/cm^3^)*	72.3 (5.5)	72.2 (5.3)	0.82	71.2 (0.9)	72.8 (2.1)	0.04*
26	Muscle area tibia 66% (mm^2^)	6322 (1759.8)	6147.8 (2033.3)	0.77	4890.2 (1436.5)	5618.8 (128)	1.00
27	Fat-to-muscle area ratio at 66%	35.4 (5.2)	35 (17.5)	0.77	69.9 (29.2)	65.5 (51.3)	0.79
28	Cortical thickness at 66% (mm)	4.0 (0.9)	4.0 (0.3)	0.91	3.4 (0.7)	3.7 (0.4)	0.18
29	Periosteal thickness at 66% (mm)	87.5 (7.9)	92.1 (10)	0.60	78.7 (4.9)	76.3 (7.7)	0.23
30	Endosteal thickness at 66% (mm)	61.8 (11.1)	65.1 (9.9)	0.49	57.5 (7.2)	53.6 (7.7)	0.05
	Handgrip strength						
31	Handgrip strength left hand average (kg)*	31 (13.2)	25.2 (10.0)	0.25	15.2 (8.4)	23.3 (14.0)	0.03*

## Discussion

In this pilot case-control study, in patients who had recovered from COVID-19 infection and had been hospitalized (average 10 days) and received steroid treatment (less than 4 mg/kg), around 40% required oxygen therapy. While there were no differences between men who had recovered from COVID-19 infection and healthy controls, we found that the muscle density, handgrip strength, and lean mass percentage were lower and fat percentage was higher among post-COVID-19 recovered female patients as compared to healthy female controls. This difference remained when parameters were compared in female patients as per their menopause. Premenopausal women who had recovered from COVID-19 infection had lower muscle parameters in comparison to healthy controls.

Recent studies have reported that over a third of infected patients with COVID-19 develop a broad spectrum of neurological symptoms affecting the central and peripheral nervous system, and skeletal muscles. COVID-19 infection has been reported to affect the musculoskeletal system, causing symptoms such as fatigue, arthralgia, myalgia, and muscle weakness, which can persist for weeks or months after the end of the infection [2].

In a case-control study conducted in Iraq from November 2021 to March 2022 in 80 post-COVID-19 recovered patients and 50 healthy controls (age of the participants: 18-60 years), the authors found significantly reduced BMD on DXA scan among post-COVID-19 recovered patients [3]. However, in the current study, there were no significant differences in BMD among post-COVID-19 recovered patients and healthy participants on DXA and pQCT scans. While we found that the T-score was lower in the post-COVID-19 recovered male and female patients at the lumbar spine and in female patients at the femur, it did not reach statistical significance. Reasons could be a shorter duration of hospitalization or the type and duration of steroid. Further, our study was cross-sectional, and a longer follow-up would possibly reveal differences in bone density between the groups.

There are no Indian studies with a design like ours. In a study from Delhi in post-recovered patients with diabetes, the authors found decreased muscle strength as measured by a hand dynamometer [12]. In a study conducted on post-COVID-19 recovered patients from the Netherlands, the authors found that participants had decreased muscle strength, a finding similar to our study; we also found that female patients had reduced strength in comparison with healthy controls [13]. In an exploratory secondary analysis of baseline data from the EXER-COVID Crossover Study (this study aims to evaluate the effectiveness of a multicomponent exercise program in improving recovery outcomes among individuals with post-discharge symptoms following COVID-19), the researchers compared post-COVID-19 recovered patients with controls within the 20-60 age group. The results revealed that post-COVID-19 recovered patients exhibited a lower percentage of lean mass, a higher percentage of fat, and decreased muscle strength in comparison to the controls [14,15]. We found similar results in our post-COVID-19 recovered female patients. Lean mass percentage and handgrip strength were significantly lower in post-COVID-19 recovered female patients than controls (median (IQR): 52% (48.3%) versus 64.6% (19.8%), p<0.05) and (median (IQR): 15.2 (8.4) kg versus 23.3 (14.0) kg, p<0.05), whereas fat percentage was higher among post-COVID-19 recovered female patients than controls (median (IQR): 44.8% (1.9%) versus 41% (6.5%), p<0.05).

The inflammatory response to SARS-CoV-2 in the respiratory tract may lead to systemic inflammation that impacts many organ systems including the musculoskeletal system. Studies have described how SARS-CoV-2 infection induces systemic elevations of cytokines and signaling molecules such as CKCL19, IFN-γ, IL-1β, IL6, IL-8, IL-17, and TNF-α. These inflammatory molecules have numerous potential mechanisms by which they may cause musculoskeletal symptoms. IFN-γ, IL-1β, IL-6, IL-17, and TNF-α are known to directly impact skeletal muscle by inducing fiber proteolysis and decreasing protein synthesis. IL-1β and IL-6 may cause fibrosis by inducing increased muscle fibroblast activity. IL-1β and TNF-α have been described to inhibit the differentiation and proliferation of satellite cells, the progenitor cells involved in muscle fiber growth. CXCL10, IL-17, and TNF-α induce osteoclast genesis and inhibit osteoblast proliferation and differentiation, causing increased bone fragility. IL-1β, IL6, and TNF-α induce chondrolysis, leading to arthralgia and/or the progression of osteoarthritis. IL-1β, IL-17, and TNF-α may contribute to tendinopathy by impairing the biological activity of tenocytes. Therefore, these inflammatory molecules may be involved in the decreased muscle strength and increased bone fragility associated with COVID-19 infection. Additionally, corticosteroids administered during hospitalization can lead to muscle atrophy, muscle weakness, and reduced bone mineral density [16].

The strength of our study is that we assessed musculoskeletal parameters as well as muscle function in patients who had suffered a serious disorder such as COVID-19 infection. We used equipment such as the DXA, pQCT, and hand dynamometer for assessments of bone mineral density, muscle density, trabecular density, cortical density, and muscle strength. However, our study has limitations, including a modest sample size, a cross-sectional design that cannot establish causation, the absence of blood markers to correlate systemic inflammation with musculoskeletal changes, and the inability to examine underlying determinants.

## Conclusions

To conclude, lean mass, muscle density, and handgrip strength were lower and body fat was higher in post-COVID-19 recovered female patients, thus indicating the need to focus on improving the musculoskeletal health of female patients post-hospitalization due to a serious illness such as COVID-19 infection. The findings of this study could aid in the development of targeted rehabilitation strategies or guidelines to support individuals recovering from COVID-19 infection, with a particular focus on female patients. Larger, longitudinal studies are required to further study the impact of the disease and treatment on the musculoskeletal health of patients who have recovered from an illness such as COVID-19 infection.

## References

[1] Pleguezuelos E, Del Carmen A, Llorensi G (2021). Severe loss of mechanical efficiency in COVID-19 patients. J Cachexia Sarcopenia Muscle.

[2] Dos Santos PK, Sigoli E, Bragança LJ, Cornachione AS (2022). The musculoskeletal involvement after mild to moderate COVID-19 infection. Front Physiol.

[3] Al-Azzawi IS, Mohammed NS (2022). The impact of angiotensin converting enzyme-2 (ACE-2) on bone remodeling marker osteoprotegerin (OPG) in post-COVID-19 Iraqi patients. Cureus.

[4] Elmedany SH, Badr OI, Abu-Zaid MH, Tabra SA (2022). Bone mineral density changes in osteoporotic and osteopenic patients after COVID-19 infection. Egypt Rheumatol Rehabil.

[5] Sapra L, Saini C, Garg B, Gupta R, Verma B, Mishra PK, Srivastava RK (2022). Long-term implications of COVID-19 on bone health: pathophysiology and therapeutics. Inflamm Res.

[6] (2020). Recommended dietary allowances and estimated average requirements -nutrient requirements for Indians. A report of the expert group of the Indian Council of Medical Research-National Institute of Nutrition. Indian Council of Medical Research. Hyderabad: National Institute of Nutrition. Indian Council of Medical Research.

[7] Ebeling PR, Nguyen HH, Aleksova J, Vincent AJ, Wong P, Milat F (2022). Secondary osteoporosis. Endocr Rev.

[8] Pal R, Aggarwal A, Singh T (2020). Diagnostic cut-offs, prevalence, and biochemical predictors of sarcopenia in healthy Indian adults: the Sarcopenia-Chandigarh Urban Bone Epidemiological Study (Sarco-CUBES). Eur Geriatr Med.

[9] Rauch F, Schöenau E (2005). Peripheral quantitative computed tomography of the distal radius in young subjects - new reference data and interpretation of results. J Musculoskelet Neuronal Interact.

[10] Rauch F, Schoenau E (2008). Peripheral quantitative computed tomography of the proximal radius in young subjects--new reference data and interpretation of results. J Musculoskelet Neuronal Interact.

[11] Ó Breasail M, Janha R, Zengin A, Pearse C, Jarjou L, Prentice A, Ward KA (2023). Cross-calibration of IDXA and pQCT scanners at rural and urban research sites in the Gambia, West Africa. Calcif Tissue Int.

[12] Mittal J, Ghosh A, Bhatt SP, Anoop S, Ansari IA, Misra A (2021). High prevalence of post COVID-19 fatigue in patients with type 2 diabetes: a case-control study. Diabetes Metab Syndr.

[13] van Gassel RJ, Bels J, Remij L (2021). Functional outcomes and their association with physical performance in mechanically ventilated coronavirus disease 2019 survivors at 3 months following hospital discharge: a cohort study. Crit Care Med.

[14] Ramírez-Vélez R, Legarra-Gorgoñon G, Oscoz-Ochandorena S (2023). Reduced muscle strength in patients with long-COVID-19 syndrome is mediated by limb muscle mass. J Appl Physiol (1985).

[15] Ramírez-Vélez R, Oteiza J, de Tejerina JM (2022). Resistance training and clinical status in patients with postdischarge symptoms after COVID-19: protocol for a randomized controlled crossover trial "The EXER-COVID Crossover Study". Trials.

[16] Hasan LK, Deadwiler B, Haratian A, Bolia IK, Weber AE, Petrigliano FA (2021). Effects of COVID-19 on the musculoskeletal system: clinician’s guide. Orthop Res Rev.

